# Supplementary material for: Sharing Data and Transferring Samples Within Pediatric Clinical Studies: How to Overcome Challenges and Make Them a Science Opportunity
Source: Healthcare (Basel). 2024 Dec 6;12(23):2473. doi: 10.3390/healthcare12232473 (PMC11641694; doi:10.3390/healthcare12232473)
Supplement: Supplementary file 1 [file healthcare-12-02473-s001.zip › EPIICAL Assent template.pdf]

INFORMATION SHEET FOR CHILD ASSENT (date, version x.x)

Title: ______________________

Sponsor's protocol code: _____________

Sponsor: _____________

Research Center: __________________________

Code of Participant: (center-number) _____________________________

Why are we doing this study?

We are interested to learn, now that you are older, how you are responding to the treatment and how your body is defending itself against illness. We also want to learn some of your family's thoughts and feelings towards treatment and research.

Why have we chosen you to take part?

We chose you because you started on treatment when you were 3 months of age or younger and have been taking treatment for at least seven years.

What will happen if you decide not to take part?

You can choose not to take part if you don't want to. You and your family will continue to get the same services care regardless of whether you take part or not.

What will happen if you decide to take part?

We will take blood from you. We need to collect approximately X teaspoons of blood from you in the next XX weeks. If you are big enough, this will be done in one visit. Otherwise, we will collect the same amount but split over up to XX visits. The amount of blood we will draw take at a single time point is based on your weight and doctors have reviewed these amounts and considered them safe.

We will also ask questions about you and your family's health, your family circumstances and review past medical records information about your health.

We will send the blood to laboratories here for some tests and some will be sent to other laboratories in <*please include the countries where samples will be sent*> where we will do additional tests to look at your health.

You can talk to us, ask questions and change your mind anytime!

Access to your medical information will be given only to authorised persons. Your data will remain secret and stored for X years. Your health is private!

The results of the study will be communicated to the scientific community through EPIICAL web portal, www.https://www.epiical.org/, congresses, scientific journals. Your identity will be always kept secret.

ASSENT FROM THE CHILD (IF AGE APPROPRIATE)

Title: ______________________

Sponsor's protocol code: _____________

Sponsor: _____________

Research Center: __________________________

Code of Participant: _____________________________

I, **(name and surname)** ………………………………………………………………………have been invited to participate in this research.

The information sheet which explains the study XX has been read to me, and I have received a copy of this sheet.

I have received enough information about the study. I understand this information and I have been able to ask questions about the study. All my questions have been answered.
I have had enough time to think carefully about taking part in this study.
I have spoken to **(name)…………………..**

I understand that it is completely up to me if I want to take part in this study and that I can stop taking part in the study at any time without providing a reason for this and return to my previous care without any disadvantage.

I understand that my medical information may need to be looked at by certain groups who have been given permission to do so and if this happens my information will be kept secret and stored for X years. I have understood that I can ask to have access to, modify or delete my information and that I can agree to or deny the further use of my data when I will become adult.

I understand that I may not be given the results of tests done on my samples that will be stored. I can receive the results of the study.

I agree to take part in the study.

Date

____

Name and surname of child (*please use capital letters*)

____

Signature of child

____

Date

____

Name and surname of parent/caregiver (*please use capital letters*)

____

Signature of parent / caregiver

____

DECLARATION BY THE RESEARCHER

I have read out the assent form in the presence of the child and he/she has had the opportunity to ask questions about the research. I also declare that the child has given his/her assent to take part freely and voluntarily and has not been coerced in any way.

A copy of this assent form has been given to the patient’s parents or legally designated representative.

Date

____

Name and surname of researcher (*please use capital letters*)

____

Signature of researcher

____
