# Supplementary material for: Sharing Data and Transferring Samples Within Pediatric Clinical Studies: How to Overcome Challenges and Make Them a Science Opportunity
Source: Healthcare (Basel). 2024 Dec 6;12(23):2473. doi: 10.3390/healthcare12232473 (PMC11641694; doi:10.3390/healthcare12232473)
Supplement: Supplementary file 1 [file healthcare-12-02473-s001.zip › EPIICAL DSA MTA template.pdf]

Material Transfer Agreement

This agreement (the “**Agreement**”) is between:

*[Provider legal name] (the “**Provider**”)*

AND

*[Recipient legal name] (the “**Recipient**”)*

hereinafter individually referred to as a “**Party**” and collectively referred to as the “**Parties**” in this Agreement.

This Agreement is aimed at regulating the sharing and the transfer among the Parties of

- Biospecimens and associated Data

from HIV-infected children in the framework of the EPIICAL 2020-2024 project (Novel Strategies to Induce Long-Term Viral Remission in Early Treated HIV Infected Children) within the study:

Title of the study: ……………..

Sponsor: …………….

Coordinating Investigator: …………..

Principal Investigator for *[Provider Legal name]*…………….. - …………..

Principal Investigator for *[Recipient Legal name]* …………….. - …………..

The current document has to be read in conjunction with the already signed EPIICAL Research Collaboration Agreement (including its attachments), the Study Agreement and the [study name/acronym] Data Sharing Agreement.

This Agreement shall come into force on the last day of signature (“Effective Date”) and shall remain in force until the final study report is released. The Sponsor may terminate, upon notification, the Agreement without delay in the case that the Provider and/or the Recipient breaches its obligations set forth in the present Agreement.
The Parties agree that the conditions of this Agreement may not be amended or modified unless in writing and signed by all the Parties. Samples and associated Data will be shared and managed in accordance with the terms of this Agreement.

Applicable Definitions

For the purpose of the document, the following definitions apply:

Biospecimen	Sample of biologic materials taken from the human body.
Biospecimen handling	Any operations performed for the receipt, storage, manipulation, archiving of the biospecimen.
Data	Personal Data information associated with the biospecimens which can be processed manually, electronically or by other means.
De-identification	Process of removing or obscuring any identifiable information from individual records in a way that minimizes the risk of unintended disclosure of the identity of individuals and information about them.
Informed consent	The agreement by a subject/legal representative(s) to participate in a research activity, by providing biospecimen and personal associated Data, after receiving adequate information and explanation about any purposes, risks and implications that the research implies. The informed consent is documented in a written form, signed and dated.
Informed assent	The legally required expression of the minor's will to participate in a clinical study dependent on the domestic law.
Provider	The registered legal entity that will provide biospecimens and associated Data to the Recipient.
Pseudonymisation	The processing of personal Data in such a manner that the personal Data can no longer be attributed to a specific Data subject without the use of additional information, provided that such additional information is kept separately and is subject to technical and organizational measures to ensure that the personal Data are not attributed to an identified or identifiable natural person.
Recipient	The registered legal entity that will receive biospecimens and associated Data from the Provider.

Part A: Declarations from Provider

The Provider **[name of organization],** located in **[address, country and contact details]** represented by **[name and surname]** in the quality of **[legal representative/principal investigator/laboratory lead/other]** hereby declares that:

1. The legal and ethical framework in force in the country is in line with the international provisions concerning medical research, in order to guarantee research quality, security and privacy protection.
2. The legal and ethical framework in force in the country allows the international transfer of biospecimens and associated Data.
3. Provided associated Data consist of the following: ***(please, specify if Data refer to outcomes of clinical/laboratory/instrumental analyses, medical records, genetic testing results, Case Report Forms, electronic Case Report Forms, others)***
   - ……
   - …….
4. The expected number of individuals providing associated Data is **[number]**
5. Provided biospecimens consist of the following: ***(please indicate the type of biological material, e.g. blood samples, tissue type, cell preparation, DNA, RNA, protein, other)***
   - ……
6. The expected number of individuals providing biospecimen is **[number]**
7. Provided associated Data have been de-identified, stripped of all information identifying individuals, without any direct means of identification.
8. De-identification measures foresee pseudonymisation (replacing names or other direct identifiers with codes or numbers).
9. Provided biospecimens have been handled and stored in compliance with the international quality standards for human biologic materials and the specific methods/measures applicable to the type of biospecimens.
10. To ensure traceability of the material, a code will be applied to the tubes. The code linking Data to the biospecimens will remain with the Provider.
11. Biospecimens will be prepared and shipped in accordance with postal regulations.
12. Shipping will be performed according to the following regulation: ………………………… (such as IATA, International Air Transport Association, and ADR, European Agreement on International Carriage of Dangerous Goods).

13. To ensure the confidentiality and security of the associated Data, transfer and processing will be safely handled and associated Data will not be transported together with biospecimens.
14. Data will be collected and stored in a password protected database and transferred from the Provider to the Recipient using password-protected CD-ROM or any other password-protected hardware system.
15. Informed consent for storage, distribution and for allowing bio-specimens and Data sharing abroad was obtained from the subject, or legal representative in case of minors, as well as informed assent where required.
16. At the end of the Agreement biospecimens may be destroyed or returned. A written notification/certification with the confirmation of the destruction/return is mandatory at the end of the Agreement and shall be sent no more than 10 days from the end of the Agreement.
17. At the end of the Agreement associated Data may be destroyed or made anonymous. A written notification/certification with the confirmation of the destruction/anonymisation is mandatory at the end of the Agreement and shall be sent no more than 10 days from the end of the Agreement.

Part B: Declarations from Recipient

The recipient **[name of organization],** located in **[address, country and contact details]** represented by **[name and surname]** in the quality of **[legal representative/laboratory lead/other to be specified]** hereby declares that:

1. The associated Data will be used solely for the scope and for the purposes and in the framework of the **[Study name/acronym]** study.
2. Biospecimens and associated Data will be received, stored and used in compliance with the applicable laws, regulations (i.e., Regulation (EU) 2016/679), policies and guidelines as well as the instructions included in the annexes of the study protocol and the Sponsor will be continuously kept informed on that use.
3. Biospecimens and associated Data will not to be transferred to anyone who is not part of the Recipient Institution / facility without the prior written consent of the Provider Institution.

4. Upon notification by the Provider, at any time, that one or more study subjects have withdrawn their consent for the continued use of their biospecimen and associated Data, the Recipient will not use anymore those parts of the Data. Biospecimens and associated Data may be destroyed or returned according to points 16-17.

Time, place and signatures of the people responsible for the institutions involved.

Signed on behalf of the Parties:

PROVIDER (specify the role) ……………………………………

……………………………………

RECIPIENT (specify the role) …………………………………

……………………………………

Declaration from the Sponsor (or delegate)

I……. in the quality of……….. (specify if delegate or legal representative) confirm that the present Declaration has been filled in and signed in accordance with the **[Study name/acronym]** study rules and responsibilities defined in the annexes of the study protocol and as defined by the Competent Authorities.

Date……. Signature………
