# Supplementary material for: Sharing Data and Transferring Samples Within Pediatric Clinical Studies: How to Overcome Challenges and Make Them a Science Opportunity
Source: Healthcare (Basel). 2024 Dec 6;12(23):2473. doi: 10.3390/healthcare12232473 (PMC11641694; doi:10.3390/healthcare12232473)
Supplement: Supplementary file 1 [file healthcare-12-02473-s001.zip › EPIICAL Informed consent template.pdf]

INFORMATION SHEET AND INFORMED CONSENT FOR PARENTS / CAREGIVERS OF PARTICIPANTS (version xx, date)

Title: ___________
Sponsor's protocol code: __________
Sponsor: __________
Research Center: _________________________
Principal Investigator in the Center: _____________________
Researcher who provides the information: _____________________
In case of emergency, contact _____________________________
Code of Participant: (center-number) ____________________________

This document will explain to you about a research study that we would like you and your child to take part in. This study has been developed by a group of African, American and European doctors and scientists.

This study has been approved by the ………… Ethics Committee. Before you decide, we would like to explain what this study is about and what it means for you and your child to join the study. You may choose to join in the whole study, or just some aspects, or in none. Please, take your time to read the information sheet and ask any question that may arise.

Why are we doing this study?

When a child has HIV, the virus gets into his/her defenses. The sooner we start children with HIV on treatment (like antiretroviral medication), the better the child will grow and develop. However, despite the treatment, some of the virus will remain hidden. This hidden virus is called the reservoir. We believe that the sooner we treat with antiretroviral medication, the smaller the reservoir will be. We, the scientists, would like to look at the virus that is part of this reservoir to see if there is anything different to the way this virus responds to certain experiments in laboratories. We want to know how the virus first responded when antiretroviral medication was started. We also want to know how the child's defense system works against HIV when treatment is started early. Finally, we want to learn some of the family's thoughts and feelings towards treatment and research.

Why have we been asked to take part?

We chose your child because he/she has HIV in his/her body, and he/she started treatment at X months of age or younger and has been on treatment for at least X years.

What will happen if you decide not to take part?

Your child's participation in the study is voluntary. It is totally up to you whether you and your child take part in this study. If you agree to participate, you will sign the consent. If you decline to participate, your child will continue with the normal standard of care treatment. Your decision will not have any influence in the management of his/her disease and your child will receive the same care if you choose not to take part in this study.

What will we need to do if your child takes part?

The participation in the study has up to X visits to the clinic during the next XX weeks. During these visits we will take blood samples from your child and ask some questions about his/her/his health, your health and family circumstances and review past medical records about the health of your child. We will also ask you questions about your thoughts and feelings towards research.

We will collect XX millilitres of blood (approximately X teaspoons) from your child. If your child is big enough, this will be done in one visit. Otherwise, we will collect the same amount but split over more than one visit. The amount of blood we will draw at a single time point is based on weight and doctors have reviewed these amounts and considered them safe.

We will send the blood to laboratories here for some tests and some will be sent to other laboratories in <*please include the countries where samples will be sent*> where we will do additional tests to look the reservoir and how the child's defenses work.
This will include genetic tests, which means how the child responds to HIV depending on his/her specific genetic characteristics.

We will also collect information from the child's and mother's clinical records to study if the amount of virus that is hidden in your child is associated with either the child's or mother's features.

We would like to store any remaining blood if you agree, to use them for research purposes. If any of these stored samples are to be transferred to another location, we will first get the permission of the …… Ethics Committee before this takes place. We will store any remaining blood samples collected as part of this study for up to X years after the completion of the study. You may contact us at any time to have your samples removed if you do not wish them to remain in storage for future tests.

In the future, if there are new findings in this area that justify new tests, we may perform some new tests using these samples. In this case, we will ask in advance for the permission of an Ethics Committee. Possibly, and if feasible, we will also contact you to inform you and get your permission before we do any new tests.

Would you like that also genetic tests are performed on your samples?

Part of the blood samples collected may be used for genetic tests. This means examining how the child responds to HIV depending on his/her specific genetic characteristics. More specifically, we will explore factors related to some processes that happen in the body of your child to control and store the HIV virus.

We don't need additional blood draws for these tests. These tests will not be able to identify your child but will only look at how their inherited genes respond to infections.

So, we would like to ask you to give consent also for these genetic tests. You may decline participation of your child in the genetic tests and still take part in the study without disadvantage or penalty.

What are the possible benefits and disadvantages of taking part?

Some tests in this study will be done immediately and others will be done later. At the end of the study, we will know if your child controls the virus well, or not. We hope that this study will provide important information to improve the treatment of HIV in the future but may not benefit your child directly.

The volume of collected blood can be easily replaced by the body it within a few days. The blood collection can, in rare cases, cause bruising, bleeding or infection at the puncture site. This can be treated immediately by the study staff. A trained nurse will do the blood collection following the standards of care and we will do our best to ensure that blood sampling does not harm your child.

Costs of the study and compensation

All tests and visits you will be receiving during the study are free of charge. If you decide to participate, you may receive your travel expenses and loss of income due to the participation in the study (up to ……), if necessary for study visits.

More information about taking part

Subject Confidentiality

We will ensure that your child's privacy is maintained, according to the European General Data Protection Regulation, Regulation (EU) 2016/679 and the <*please include the national/local*

laws>. Your child will be identified only by a code consisting in a subject number. All documents will be kept in a strictly confidential file by the investigators. His/her identity will not be revealed to any person but to the authorized personnel participating in the study. Researchers will have access to your clinical record and responsible individuals designated by the Sponsor may be given data access for monitoring and/or audit to ensure that the study complies with regulations. Data will be treated confidentially, according to European General Data Protection Regulation, Regulation (EU) 2016/679 and the <*please include the national/local laws*>. Any identifiable personal information will be stored and processed under safe conditions of safety.

You have the right to refuse or to withdraw from the study at any time without giving a reason for the decision and to ask for the removal of all samples collected unless not yet analysed. No further data besides that essential for the study will be collected. Data collected will remain in the study records.

What will be done with the results?

The results of the study will be communicated to the scientific community through the EPIICAL web portal, www.https://www.epiical.org/, congresses and scientific journals. This will not identify you personally, but you will be in the groups described. Anonymous data from this study will be made publicly available so that other researchers can check that our research is right and to answer other scientific questions. You will not be able to withdraw your consent for the sharing of data that has already been collected. You will not benefit personally from these extra studies. Your identity and your child's will be always maintained confidential, only the doctor and scientists participating in the study will have access to any data that can be linked individually to your child. In the event of unexpected or incidental findings, that is findings discovered in the course of conducting research but beyond the aims of the study, the investigators will disclose this information to you, if you agree and if any treatment or preventive measures is available.

Who has checked that this study is safe?

This study has been reviewed by an independent group of people (an Ethics Committee). Their job is to make sure that the study has been planned in a way to protect the rights, safety and wellbeing of participants.

Who is organizing this study?

This study is organized by ________________and funded by ________________.

Any question that arises after reading this document should be addressed to:

Dr. ________________________(site PI)

Hospital ________________________

Department of __________________

Address_________________________

Telephone_______________________

You will have a signed and dated copy of this document and another copy will be kept in the medical records of this clinical site.

In case you wish to ask any question to someone else about this study, please contact:

National Bioethics Committee in Health/Ethics Committee/Institutional Review Board

Address____________________

Telephone________________

CONSENT FROM PARENT (>18 y) OR LEGAL REPRESENTATIVE OF THE CHILD

Title: _____________

Sponsor: _____________________

Research Center: ________________________

Code of Participant: _____________

I, **(name and surname)** ………………………………………………… ………………..

as **(relationship with the Participant)** ………………………….........................

of **(participant's name)** ……………………………………………………………...

I have read the information sheet (version ………, date………..) provided relating the study XX, and I have received a copy of this document.

I have received enough information about the study. I have been able to make questions about the study, and I have received satisfactory answers to my questions.

I have had enough time to thoroughly and fully consider my child's participation in the study.

I have spoken to **(name) …………………..**

I understand that our participation is voluntary.

I understand that I have the right to refuse or to withdraw from the study at any time without providing a reason for my decision and return to the standard care without any penalty or loss of benefits.

I understand that clinical information may be reviewed by properly authorised individuals as part of the study and that such information will be treated as strictly confidential.

I understand that my child and I may not be given the results of tests performed on stored samples.

Tick according to parent/legal guardian's consent:

Allow blood samples (XX teaspoons) to be taken from my child in up to X visits in the next XX weeks, to be tested for the study purpose, and to be sent to a laboratory both in this Country and out of my Country	☐ YES<br>☐ NO
Allow researchers to access my child's clinical record data	☐ YES<br>☐ NO
Allow my child's samples to be stored up to X years after completion of this study for possible later research studies similar to this study	☐ YES<br>☐ NO
Allow routine blood results and clinical information being included coded/pseudonymised in a database for the study	☐ YES<br>☐ NO
Allow aggregated and anonymised (identity of child is protected) study data to be communicated or published in specialized reports	☐ YES<br>☐ NO
Agree to know possible unexpected or incidental findings and if any treatment or preventive measures is available	☐ YES<br>☐ NO
Agree to be recontacted for future research studies	☐ YES<br>☐ NO

I agree to my child's participation in the study:

Date

____

Name and surname of parent/caregiver (*please use capital letters*)

____

Signature of parent / caregiver

____

Date

____

Name and surname of researcher (*please use capital letters*)

____

Signature of researcher

____

Genetics:

My child's samples to be used for genetic tests	☐ YES<br>☐ NO

Date

____

Name and surname of parent/caregiver (*please use capital letters*)

____

Signature of parent / caregiver

____

Date

____

Name and surname of researcher (*please use capital letters*)

____

Signature of researcher

____

Allow researchers to access my clinical record data	☐ YES<br>☐ NO

In the event that parent/legal guardian is unable to read the consent form:

Name and Signature of the person who read and explained the consent form:

____

I ____certify that all the information related with this study has been clearly explained and apparently understood by the parent/caregiver of the child and that the consent has been freely given by the parent/caregiver of the child.

Date: _____/_____/_____

Witness Name (*please use capital letters*):

____

Witness Signature*:

____

*A witness is a person who is independent from the research team or any team member and who was not involved in obtaining consent

Digital impression of the parent/caregiver:

Mother/Father/Caregiver Contact:

Name____

Contact____
